# Supplementary material for: Service quality, satisfaction, and intention to use Pourasava Digital Center in Bangladesh: The moderating effect of citizen participation
Source: PLoS One. 2024 Jun 12;19(6):e0304178. doi: 10.1371/journal.pone.0304178 (PMC11168632; doi:10.1371/journal.pone.0304178)
Supplement: Attachment — Submitted filename: Comments to the Author.docx [file pone.0304178.s002.docx]

**Comments to the Authors**

In this paper, the author proposes a study of Service Quality and Satisfaction of Pourasava Digital Center (PDC) in Bangladesh. Overall, the paper demonstrates a solid understanding of the subject matter but could be improved by providing a clearer statement of the research problem in the introduction, a more critical analysis in the literature review, and a more detailed rationale for the hypotheses. These enhancements would contribute to a more robust and compelling research paper.

1. **Introduction:** The introduction sets the stage for the study but could benefit from a more detailed exposition of the research context. Specifically, the last paragraph lacks a clear articulation of the research gap that this study aims to address. Enhancing this section with a concise statement of the research problem and its significance would strengthen the foundation of the paper.
2. **Literature Review:** The literature review appears comprehensive but somewhat lacks critical analysis. The authors might consider not only summarizing existing studies but also critically evaluating them, highlighting contradictions or gaps in the literature. Additionally, providing clear explanations for the choice of constructs in the proposed model would be advantageous for the readers. This would better position their study within the existing body of knowledge and justify the need for their research.
3. **Theoretical Framework and Hypotheses Development:** The theoretical framework is well-constructed, integrating relevant theories effectively. However, the development of hypotheses seems to be more descriptive & brief than analytical & comprehensive. It would be beneficial to provide a more in-depth rationale for each hypothesis, linking them more explicitly to the theoretical framework. This would enhance the reader's understanding of how the hypotheses are grounded in the theory.
4. **Statistical Analysis:** The statistical analysis is comprehensive, but the interpretation of some model fit indices (e.g., RMSEA of .149) (mentioned in Section 5.3 Hypotheses Testing) suggests potential issues with model fit. A RMSEA value of .149 is generally considered high, indicating a poor fit. The authors should address this in their discussion, considering either model re-specification or providing a rationale for the acceptance of these fit indices. Additionally, the rationale behind accepting or rejecting specific hypotheses should be clearly articulated, especially in cases where the results are marginal or counterintuitive. The following information may be helpful for the authors:

- RMSEA ≤ 0.05: Indicates a close fit of the model in relation to the degrees of freedom.
- RMSEA between 0.05 and 0.08: Suggests a reasonable error of approximation in the population.
- RMSEA between 0.08 and 0.10: Represents a mediocre fit.
- RMSEA > 0.10: Is considered a poor fit.

5. **Discussion:** The discussion effectively connects study results with previous research, but it could benefit from deeper analysis of why certain hypotheses were not supported. Specifically, exploring potential reasons for the unexpected findings (e.g., H1a, H3a, H7) would provide a more comprehensive understanding and contribute to the field's knowledge.
6. **Research Implications:** The implications are broadly stated but lack specificity in how the findings can be practically applied. Detailing concrete steps for PDC improvement based on the study's results would enhance the practical utility of the research.

while the paper offers valuable insights into the Service Quality and Satisfaction at PDC in Bangladesh, refining its research problem articulation, deepening analytical rigor, and addressing statistical concerns will significantly enhance its scholarly and practical contributions.

Top of Form
