# Supplementary material for: Service quality, satisfaction, and intention to use Pourasava Digital Center in Bangladesh: The moderating effect of citizen participation
Source: PLoS One. 2024 Jun 12;19(6):e0304178. doi: 10.1371/journal.pone.0304178 (PMC11168632; doi:10.1371/journal.pone.0304178)
Supplement: Attachment — Submitted filename: Response to Reviewers 1st Revision.docx [file pone.0304178.s003.docx]

**Responses to Reviewer**

**Comments to the Author**

1. Is the manuscript technically sound, and do the data support the conclusions?

The manuscript must describe a technically sound piece of scientific research with data that supports the conclusions. Experiments must have been conducted rigorously, with appropriate controls, replication, and sample sizes. The conclusions must be drawn appropriately based on the data presented.

Reviewer #1: Yes

Reviewer #2: Yes

2. Has the statistical analysis been performed appropriately and rigorously?

Reviewer #1: Yes

Reviewer #2: Yes

3. Have the authors made all data underlying the findings in their manuscript fully available?

The [PLOS Data policy](http://www.plosone.org/static/policies.action#sharing) requires authors to make all data underlying the findings described in their manuscript fully available without restriction, with rare exception (please refer to the Data Availability Statement in the manuscript PDF file). The data should be provided as part of the manuscript or its supporting information, or deposited to a public repository. For example, in addition to summary statistics, the data points behind means, medians and variance measures should be available. If there are restrictions on publicly sharing data—e.g. participant privacy or use of data from a third party—those must be specified.

Reviewer #1: Yes

Reviewer #2: Yes

4. Is the manuscript presented in an intelligible fashion and written in standard English?

PLOS ONE does not copyedit accepted manuscripts, so the language in submitted articles must be clear, correct, and unambiguous. Any typographical or grammatical errors should be corrected at revision, so please note any specific errors here.

Reviewer #1: Yes

Reviewer #2: No

**Authors’ justification:**

Thank you for your wonderful suggestions. I have revised my manuscript and solve the grammatical errors according to the review comments

5. Review Comments to the Author

Please use the space provided to explain your answers to the questions above. You may also include additional comments for the author, including concerns about dual publication, research ethics, or publication ethics. (Please upload your review as an attachment if it exceeds 20,000 characters)

Reviewer #1: In this paper, the author proposes a study of Service Quality and Satisfaction of Pourasava Digital Center (PDC) in Bangladesh. Overall, the paper demonstrates a solid understanding of the subject matter but could be improved by providing a clearer statement of the research problem in the introduction, a more critical analysis in the literature review, and a more detailed rationale for the hypotheses. These enhancements would contribute to a more robust and compelling research paper.

**Author’s Response to Reviewer #1: Thank you so much for your comments. I have revised accordingly through giving some new critical insights.**

1. Introduction: The introduction sets the stage for the study but could benefit from a more detailed exposition of the research context. Specifically, the last paragraph lacks a clear articulation of the research gap that this study aims to address. Enhancing this section with a concise statement of the research problem and its significance would strengthen the foundation of the paper.

**Author’s Response to Introduction (Reviewer #1): I have revised accordingly by analyzing a clear research gap.**

2. Literature Review: The literature review appears comprehensive but somewhat lacks critical analysis. The authors might consider not only summarizing existing studies but also critically evaluating them, highlighting contradictions or gaps in the literature. Additionally, providing clear explanations for the choice of constructs in the proposed model would be advantageous for the readers. This would better position their study within the existing body of knowledge and justify the need for their research.

**Author’s Response to Literature Review (Reviewer #1): Thank you for your excellent suggestions. I have revised accordingly and provided a critical arguments based on the literature and tried to set a gap.**

3. Theoretical Framework and Hypotheses Development: The theoretical framework is well-constructed, integrating relevant theories effectively. However, the development of hypotheses seems to be more descriptive & brief than analytical & comprehensive. It would be beneficial to provide a more in-depth rationale for each hypothesis, linking them more explicitly to the theoretical framework. This would enhance the reader's understanding of how the hypotheses are grounded in the theory.

**Author’s Response to Theoretical Framework and Hypotheses Development (Reviewer #1): Thank you for your excellent suggestions. I have updated and provided an in-depth rationale in hypotheses development and theoretical understandings.**

4. Statistical Analysis: The statistical analysis is comprehensive, but the interpretation of some model fit indices (e.g., RMSEA of .149) (mentioned in Section 5.3 Hypotheses Testing) suggests potential issues with model fit. A RMSEA value of .149 is generally considered high, indicating a poor fit. The authors should address this in their discussion, considering either model re-specification or providing a rationale for the acceptance of these fit indices. Additionally, the rationale behind accepting or rejecting specific hypotheses should be clearly articulated, especially in cases where the results are marginal or counterintuitive. The following information may be helpful for the authors:
¬ RMSEA ≤ 0.05: Indicates a close fit of the model in relation to the degrees of freedom.
¬ RMSEA between 0.05 and 0.08: Suggests a reasonable error of approximation in the population.
¬ RMSEA between 0.08 and 0.10: Represents a mediocre fit.
¬ RMSEA > 0.10: Is considered a poor fit.

**Author’s Response to Statistical Analysis (Reviewer #1): Thank you so much for your comments and for you concern I have revised accordingly.**

5. Discussion: The discussion effectively connects study results with previous research, but it could benefit from deeper analysis of why certain hypotheses were not supported. Specifically, exploring potential reasons for the unexpected findings (e.g., H1a, H3a, H7) would provide a more comprehensive understanding and contribute to the field's knowledge.

**Author’s Response to Discussion (Reviewer #1): I have revised accordingly as you suggested.**

6. Research Implications: The implications are broadly stated but lack specificity in how the findings can be practically applied. Detailing concrete steps for PDC improvement based on the study's results would enhance the practical utility of the research.

**Author’s Response to Research Implications (Reviewer #1): I have revised.**

While the paper offers valuable insights into the Service Quality and Satisfaction at PDC in Bangladesh, refining its research problem articulation, deepening analytical rigor, and addressing statistical concerns will significantly enhance its scholarly and practical contributions.

**Author’s Response: Thank you so much for your valuable comments which helped me to improve my manuscripts in many important areas.**

Reviewer #2: Dear author although topic of research is interesting how need improvements for example scope of this study is limited to one country, why you do not add other countries studies conducted in same region and enhance scope of your research for example "Investigating the role of diffusion of innovation theory in determining citizen’s intention towards adoption of e-government services" this study is also conducted in south Asia context and relevant to discuss
Improvements are required in your methodology section information regarding research paradigm is missing it is highly recommended to design your research under a specific research paradigm and you may grab this information through "Research design and methods: A systematic review of research paradigms, sampling issues and instruments development" similarly support scale adoption with latest research "Integration of DeLone and McLean and self-determination theory in internet banking continuance intention context" for information and system quality scale. you are requested to improve research conclusion it is too short and not justifiable

**Author’s Response to (Reviewer #2): I have revised based on your precise comments and updated my paper accordingly. Thank you.**
