# Supplementary material for: Service quality, satisfaction, and intention to use Pourasava Digital Center in Bangladesh: The moderating effect of citizen participation
Source: PLoS One. 2024 Jun 12;19(6):e0304178. doi: 10.1371/journal.pone.0304178 (PMC11168632; doi:10.1371/journal.pone.0304178)
Supplement: Attachment — Submitted filename: Response to Reviewers.docx [file pone.0304178.s005.docx]

**Responses to Reviewer and Academic Editor**

**Comments to the Author**

1. If the authors have adequately addressed your comments raised in a previous round of review and you feel that this manuscript is now acceptable for publication, you may indicate that here to bypass the “Comments to the Author” section, enter your conflict of interest statement in the “Confidential to Editor” section, and submit your "Accept" recommendation.

Reviewer #1: All comments have been addressed

Reviewer #2: All comments have been addressed

**Authors’ justification** Reviewer #1 & #2**:** Thank you for your comments that gave us confidence, we have tried our best.

2. Is the manuscript technically sound, and do the data support the conclusions?

The manuscript must describe a technically sound piece of scientific research with data that supports the conclusions. Experiments must have been conducted rigorously, with appropriate controls, replication, and sample sizes. The conclusions must be drawn appropriately based on the data presented.

Reviewer #1: Yes

**Authors’ justification** Reviewer #1**:** Thank you so much for your appreciating response that gave us confidence, we have tried our best.

Reviewer #2: No

**Authors’ justification** Reviewer #2**:** Thank you so much for your response in this regard that helped us to improve our manuscript regarding making a strong linkage between data and conclusion of the manuscript. We have updated according to your suggestions you made here.

3. Has the statistical analysis been performed appropriately and rigorously?

Reviewer #1: Yes

**Authors’ justification** Reviewer #1**:** Thank you so much for your appreciating response that gave us confidence, we have tried our best.

Reviewer #2: No

**Authors’ justification** Reviewer #2**:** Thank you so much for your response. We think we applied the correct statistical tool to analyze the data and tried best to analyze them in appropriate manner.

4. Have the authors made all data underlying the findings in their manuscript fully available?

The PLOS Data policy requires authors to make all data underlying the findings described in their manuscript fully available without restriction, with rare exception (please refer to the Data Availability Statement in the manuscript PDF file). The data should be provided as part of the manuscript or its supporting information, or deposited to a public repository. For example, in addition to summary statistics, the data points behind means, medians and variance measures should be available. If there are restrictions on publicly sharing data—e.g. participant privacy or use of data from a third party—those must be specified.

Reviewer #1: Yes

Authors’ justification to Reviewer #1: Thank you for your comment, we already made all the data used in this study available.

Reviewer #2: No

Authors’ justification to Reviewer #2: Thank you for your comment, but we have published all the data used in this study to the journal through submitting data file as supporting documents.

5. Is the manuscript presented in an intelligible fashion and written in Standard English?

PLOS ONE does not copyedit accepted manuscripts, so the language in submitted articles must be clear, correct, and unambiguous. Any typographical or grammatical errors should be corrected at revision, so please note any specific errors here.

Reviewer #1: Yes

Authors’ justification to Reviewer #1: Thank you for your comment.

Reviewer #2: No

Authors’ justification to Reviewer #2: Thank you for your comment that helped us to look over our manuscript and we gone through our paper and updated English language, after that the manuscript is revised by an English language expert for ensuring Standard English.

6. Review Comments to the Author

Please use the space provided to explain your answers to the questions above. You may also include additional comments for the author, including concerns about dual publication, research ethics, or publication ethics. (Please upload your review as an attachment if it exceeds 20,000 characters)

Reviewer #1: (No Response)

Reviewer #2: Dear authors unfortunately you have not address issues raised by reviewer research paradigm information is still missing and even article is not structured accordingly

**Author’s Response to Reviewer #2: Thank you so much for your feedback. We have updated our manuscript based on your feedback.**

"Reviewer #2: Dear author although topic of research is interesting how need
improvements for example scope of this study is limited to one country, why you do not
add other countries studies conducted in same region and enhance scope of your
research for example "Investigating the role of diffusion of innovation theory in
determining citizen’s intention towards adoption of e-government services" this study is
also conducted in south Asia context and relevant to discuss
Improvements are required in your methodology section information regarding research
paradigm is missing it is highly recommended to design your research under a specific
research paradigm and you may grab this information through "Research design and
methods: A systematic review of research paradigms, sampling issues and instruments
development" similarly support scale adoption with latest research "Integration of
DeLone and McLean and self-determination theory in internet banking continuance
intention context" for information and system quality scale. You are requested to
improve research conclusion it is too short and not justifiable"

**Author’s Response to Reviewer #2: Thank you so much for your rigorous feedback which helped us to improve our manuscript scientifically and theoretically sound. We have updated our manuscript based on your comments.**
